# Theranostic Nanoseeds for Efficacious Internal Radiation Therapy of Unresectable Solid Tumors

**DOI:** 10.1038/srep20614

**Published:** 2016-02-08

**Authors:** Sina Moeendarbari, Rakesh Tekade, Aditi Mulgaonkar, Preston Christensen, Saleh Ramezani, Gedaa Hassan, Ruiqian Jiang, Orhan K. Öz, Yaowu Hao, Xiankai Sun

**Affiliations:** 1 Department of Materials Science and Engineering, University of Texas at Arlington, TX 76019, USA; 2 Department of Radiology, University of Texas Southwestern Medical Center, Dallas, TX 75390, USA; 3 Advanced Imaging Research Center, University of Texas Southwestern Medical Center, Dallas, TX 75390, USA

## Abstract

Malignant tumors are considered “unresectable” if they are adhere to vital structures or the surgery would cause irreversible damages to the patients. Though a variety of cytotoxic drugs and radiation therapies are currently available in clinical practice to treat such tumor masses, these therapeutic modalities are always associated with substantial side effects. Here, we report an injectable nanoparticle-based internal radiation source that potentially offers more efficacious treatment of unresectable solid tumors without significant adverse side effects. Using a highly efficient incorporation procedure, palladium-103, a brachytherapy radioisotope in clinical practice, was coated to monodispersed hollow gold nanoparticles with a diameter about 120 nm, to form ^103^Pd@Au nanoseeds. The therapeutic efficacy of ^103^Pd@Au nanoseeds were assessed when intratumorally injected into a prostate cancer xenograft model. Five weeks after a single-dose treatment, a significant tumor burden reduction (>80%) was observed without noticeable side effects on the liver, spleen and other organs. Impressively, >95% nanoseeds were retained inside the tumors as monitored by Single Photon Emission Computed Tomography (SPECT) with the gamma emissions of ^103^Pd. These findings show that this nanoseed-based brachytherapy has the potential to provide a theranostic solution to unresectable solid tumors.

Highly localized radiotherapy with isotopes has become a standard treatment option for many unresectable solid cancerous tumors. To confine the radiation to tumor sites, two approaches are currently in clinical practice: systemic radioisotope therapy using targeted radiopharmaceuticals and brachytherapy using sealed radioactive sources. In brachytherapy, radioactive sources are surgically placed into or next to the tumor volume, whereby the radiation dose is delivered continuously, either over a short period of time (temporary implants) or over the lifetime of the source to a complete decay (permanent implants). The seeds for permanent implants are usually prepared by sealing a low dose radioactive source such as ^103^Pd (t_1/2_ = 17 d; 100% EC; E_X-ray_ = 21 keV) and ^125^I (t_1/2_ = 59.4 d; 100% EC; E_X-ray_ = 35 keV) in a metal (e.g. titanium) container with the size about 5 mm long and 1 mm wide. Brachytherapy effectively confines the therapeutic radiation dose to the tumor region while sparing normal tissues. However, the surgical implantation of millimeter size brachytherapy seeds, commonly used for the treatment of prostate cancer^1^,^2^,^3^,^4^,^5^, causes many adverse side effects and greatly limits its applications^1^. Moreover, due to the millimeter size of the seeds, following the surgical implantation a majority of patients would experience post-treatment symptoms ranging from adverse side effects to severe clinical complications^2^,^4^,^6^,^7^,^8^,^9^. With the rapid development of nanoscience and nanotechnology, it becomes appealing to make injectable nano-scale brachytherapy seeds. Because much smaller needles can be used for injection of nanoseed colloidal solution, nanoseed-based internal radiation therapy can reduce the trauma caused by surgical implantation. Also, it can provide an easy way to differentiate the radiation dosage by inject different amount of the nanoseed solution. Currently, the differentiation is only achieved by controlling the distance from the implant. More importantly, nanoseeds-based brachytherapy could dramatically expand its applications. It would enable the treatment of much smaller tumors and the procedure can be performed intraoperatively when optimal surgical resection is not possible. On the other hand, the size of nanoseeds shall be maintained reasonably large so as to prevent these radioactive particles from diffusing off the target^10^. Herein we report a Cu-mediated surface modification process to efficiently incorporate radioactive isotopes to gold nanoparticles from a solution with trace amounts (~10^−8 ^M) of isotopes. Using this method, ^103^Pd was incorporated onto hollow gold nanoparticles (~120 nm in diameter). The resulting ^103^Pd@Au nanoseeds in the form of a colloidal suspension were administered by direct injection into a prostate cancer xenograft model. The size of the nanoseed is large enough to prevent nanoseeds from diffusing into other areas, resulting in >95% nanoseeds being retained inside the tumor over the entire course of 5-week treatment. A high therapeutic efficacy was observed without noticeable side effects on the liver, spleen and other organs, which are direct results of such high retention rate inside the tumor. These results show promising potential for such therapeutic agents to become a clinically practical treatment modality for unresectable solid cancers.

## Results and Discussions

### Synthesis of ^103^Pd@Au nanoseeds

The preparation process of the ^103^Pd@Au nanoseeds starts with the synthesis of hollow Au nanoparticles using the bubble template synthesis method developed in our lab^11^,^12^. In this method, electrochemically evolved hydrogen nanobubbles serve as templates and reduce Au complex ions into metal Au. Metal Au covers the bubbles to form hollow Au nanoparticles. Anodic aluminum oxide (AAO) membrane with 300 nm diameter through channels was used inside the solution to collect nanoparticles. This synthesis method can produce monodispersed Au nanoparticles with a diameter larger than 100 nm. It is a surfactant-free process that provides a clean surface for further coating process. Synthesized Au nanoparticles feature a sub-25 nm polycrystalline shell with a 50 nm hollow core. This hollow structure also presents an additional advantage for nanoseeds due to its high specific surface area and low density.

**Figure 1:**
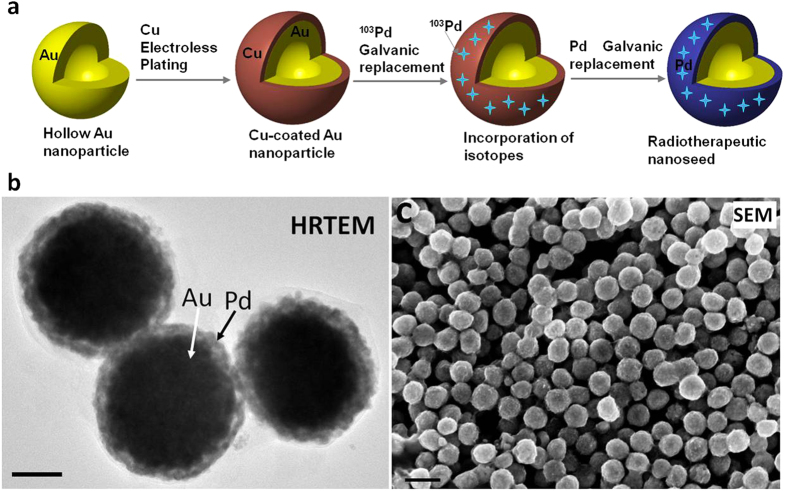
Synthesis of ^103^Pd@Au nanoseeds. (**a**) Schematic of the process of incorporation of ^103^Pd on hollow Au nanoparticles. A Cu layer was first coated by an electroless deposition process. Then, the Cu layer is replaced by Pd through a galvanic replacement reaction. (**b**) High resolution TEM micrograph of Pd@Au nanoparticles. Core-shell configuration (distinguished by their Z-contrast) is clearly shown. The particle size is around 150 nm. (**c**) SEM micrographs of Pd@Au nanoparticles showing a narrow size distribution. The scale bars in (**b**,**c**) are 50 nm and 200 nm, respectively.

To coat the Au nanoparticles with Pd, a two-step process was developed as shown in Fig. 1a^13^,^14^,^15^. The hollow Au nanoparticles were first coated with Cu by an electroless deposition process. Then, the Cu layer was partially (about 70%) replaced by Pd through a galvanic reaction. The TEM image in Fig. 1b clearly demonstrates the core-shell configuration (distinguished by their Z-contrast) with a typical size around 150 nm. The SEM image in Fig. 1c indicates an almost perfect spherical shape and a very narrow size distribution.

To prepare ^103^Pd@Au nanoseeds, a solution containing ^103^Pd was added after the electroless plating of Cu. The plating of ^103^Pd, which is the displacement reaction of the element Cu layer by [^103^Pd]PdCl_2_, was allowed to continue for 24 h upon the addition of Pd plating solution for 1 h. The incorporation rate of ^103^Pd was >80% under the condition of 1462 ± 130 μCi of ^103^Pd onto 66.8 μmol of Au (see Method section). The total synthesis time of ^103^Pd@Au nanoseeds was approximately 26 h. The ^103^Pd@Au nanoseeds were found to be extremely stable and retain their original size even after being shelved for 2 months at 8 ± 2 °C. Although caking of ^103^Pd@Au nanoseeds was observed during the storage, it can be reconstituted with phosphate-buffered saline (PBS) uniformly by mild sonication for 30 seconds. The ^103^Pd@Au nanoseeds were highly negatively charged (ζ-potential: −25.81 ± 1.8 mV), which we believe imparts the long shelf-life and stability to the particles.

The electroless deposition of Cu on Au surface is a simple and robust process. Copper can be readily replaced by less reactive metals through a single displacement reaction in an aqueous solution containing metal ions without any other additives. This Cu-mediated displacement process provides a facile way to efficiently incorporate the tracer level of radioisotopes onto Au nanoparticle surface. In addition to ^103^Pd, we were able to synthesize nanoseeds with other therapeutic radioisotopes that are chemically less reactive than Cu, such as ^198^Au and ^105^Rh. Therapeutic Cu isotopes (e.g. ^64^Cu and ^67^Cu) can also be incorporated. These isotopes are β-emitters. Compared with photon (gamma/X-ray) emissions, β−particles have much shorter radiation ranges, and are used in systemic radioisotope therapy. To date, β-emitters have not been utilized for brachytherapy because radioactive sources are encapsulated and only gamma/X-ray can penetrate the capsules. Given that the encapsulation is no longer needed, our nanoseed-based approach opens up the possibility to use β-emitters for brachytherapy.

### Animal studies

Severe combined immunodeficiency (SCID) mice bearing human prostate cancer tumors were used for *in vivo* evaluation of the nanoseeds, including their retention in tumor sites, toxicity, and therapeutic efficacy. Tumor induction was done by following methods reported earlier with slight modifications^16^,^17^. The cell suspension containing 3 × 10^6 ^PC3 cells was implanted subcutaneously into both shoulders of SCID mice. Tumors were allowed to grow for 4 weeks to reach a palpable size (~181.7 ± 62.1 mm^3^). Animals were randomized at day 0 into three groups (n = 6) to be treated with PBS solution, cold Pd@Au nanoparticles in PBS, and nanoseeds in PBS suspension, respectively. The injection was performed intratumorally at 6–9 locations each tumor so as to achieve an even distribution of the radiation dose in the whole tumorous mass. The injected radioactivity of ^103^Pd@Au nanoseeds was 1.5 mCi per tumor. The injected volume was maintained under 40 μL for all three groups: PBS only, cold Pd@Au nanoparticles, and nanoseeds. The gold nanoparticle concentration in each injection was maintained at 2.03 × 10^10 ^nanoparticles/mL.

### Retention of ^103^Pd@Au nanoseeds in tumor sites

**Figure 2:**
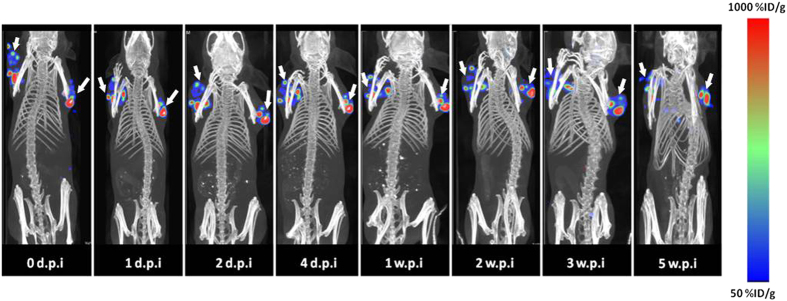
Retention of the injected ^103^Pd@Au nanoseeds at tumor sites. Serial SPECT/CT imaging performed 0, 1, 2, 4, 7, 14, 21, and 35 days post intratumoral injection of 1.51 mCi ^103^Pd@Au nanoseeds in PC3-tumor bearing SCID mice. White arrows indicate tumors.

For the nanoseed treated group, small animal SPECT/CT imaging was conducted after the intratumoral injection in a longitudinal manner (at 0, 1, 2, 4 days post injection (d.p.i.) and 1, 2, 4, and 5 weeks post injection (w.p.i.)) to noninvasively monitor the retention of the nanoseeds by acquisition of the low energy X-ray emissions of ^103^Pd on a Bioscan NanoSPECT/CT Plus System (Fig. 2a). The quantitative SPECT analysis performed at 1 d.p.i. clearly showed the injected dose stayed at the site of administration (101.50 ± 23.72% ID/g) with negligible amounts of radioactivity observed in the liver (0.11 ± 0.06% ID/g) and spleen (0.14 ± 0.01% ID/g), which are the major sites for uptake and deposition of Au nanoparticles^18^. As the study was progressing, the nanoseed uptake level in tumor noninvasively measured by SPECT increased gradually to 274.48 ± 77.62% ID/g at 5 w.p.i. concomitantly as the tumor volume shrank, indicative of the anticipated radiotherapeutic effect of the nanoseeds.

The biodistribution of the nanoseeds were further investigated by a parallel *ex vivo* assay in the same tumor model (10 mice). At 1 d.p.i. and 1 w.p.i., three mice were sacrificed; at 3 and 5 w.p.i., two sacrificed. The organs of interest (blood, heart, lung, muscle, bone, fat, liver, spleen, kidney, stomach, small intestine, large intestine, brain, and tumor) were excised, weighed, and then measured for radioactivity by a γ-counter. Thereafter, the tissues were dissolved in aqua regia to prepare samples for the analysis of inductively coupled plasma mass spectrometry (ICP-MS) to measure the Au and Pd contents. A good agreement was found between the γ-counter and ICP-MS results (no statistically significant difference, p = 0.88), indicating that the radioactive isotopes of ^103^Pd stayed with the nanoseeds during the five weeks of therapeutic study. The *ex vivo* biodistribution study demonstrated that 95.19 ± 0.94% of the nanoseeds remained within the tumor, while the rest was found in the liver (3.31 ± 1.11%) and spleen (0.39 ± 0.24%). No meaningful uptake was observed in other tissues. Evidently, the tumor uptake remained virtually the same over the five weeks of the study. (See supplemental materials for details).

The radioactive nature of brachytherapy requires a high retention rate of the dose within the administered diseased sites. The observed high retention of nanoseeds in tumor sites meets this essential requirement, showing the promising potential of our approach for clinical translation. Because these nanoseeds are not functionalized, such a remarkably high retention rate likely results from their relatively large sizes (~150 nm). It has been well documented that a majority of gold nanoparticles after intravenous injection accumulate in the mononuclear phagocyte system organs (e.g., liver and spleen) if their sizes are bigger than 10 nm^18^. In this work, however, a negligible amount of nanoseeds was found in the liver and spleen, indicating that no appreciable diffusion of nanoseeds into the blood stream occurred.

### Toxicity of ^103^Pd@Au nanoseeds

Toxicity of ^103^Pd@Au nanoseeds was assessed and compared with that of cold Pd@Au nanoparticles in the three groups of mice over the 5-week treatment period by complete blood count (CBC), alanine transaminase (ALT), aspartate transaminase (AST), blood urea nitrogen (BUN) and creatinine levels at 10 and 30 d.p.i. The red blood cell (RBC) count along with the mean hemoglobin volume per RBC (MCH) was unaffected throughout the study suggesting the nonhemolytic nature of the nanoseeds. An initial reduction of the white blood cell (WBC) count was found in the group of ^103^Pd@Au nanoseeds at 10 d.p.i. This may be due to the exposure of the shoulder girdle (marrow-producing bone) to the radiation from nanoseeds inside the tumors. The complete recovery of the WBC count was observed within 30 d.p.i. Similar observations were noted for platelet counts and other parameters. No notable differences or changes were measured for the BUN, ALT, AST, or creatinine level among the three groups of mice indicating no appreciable kidney or liver toxicity was associated with the nanoseeds. (See supplemental materials for details).

We believe that the observed low – no toxicity of the nanoseeds arises from the high tumoral retention rate, which confines the radiation damage within the tumor. This is a highly desirable feature of radiation therapy. Indeed, we further found that the nanoseeds remained in the physical location of tumors even after the tumor masses virtually disappeared in two mice of the treatment group of ^103^Pd@Au nanoseeds.

### Therapeutic efficacy of ^103^Pd@Au Nanoseeds

**Figure 3:**
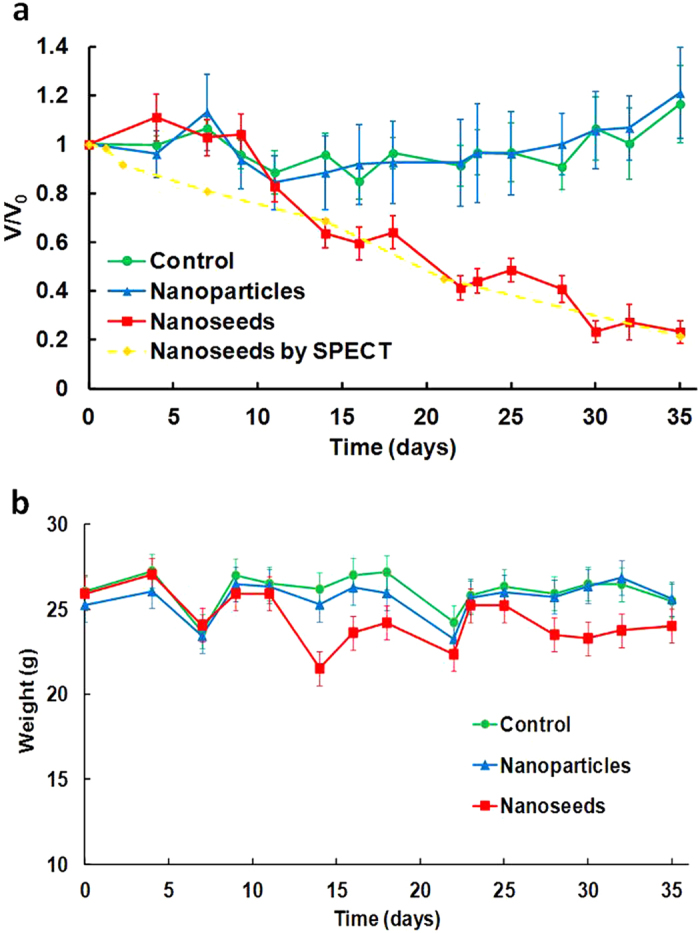
(**a**) Tumor volume and (**b**) body weight change during the progression of therapy.

Tumor volumes (Fig. 3a) in the three groups of tumor-bearing mice were measured using a caliper every other day in a double-blinded manner. After 15 d.p.i, a clear separation of tumor growth profile was observed (p < 0.0001). As expected, over the 5-week treatment period, the group treated with ^103^Pd@Au nanoseeds showed a significantly retarded tumor growth or tumor size shrinkage (82.75 ± 46.25 mm^3^ to 19.83 ± 20.12 mm^3^; p < 0.001), while the tumors continued their growth in both PBS and cold Pd@Au nanoparticle treated groups (PBS: 67.08 ± 30.96 mm^3^ to 187 ± 80.11 mm^3^; cold Pd@Au nanoparticles: 58.75 ± 35.29 mm^3^ to 122.14 ± 4.082 mm^3^). It is noteworthy that two mice in the nanoseed treated group were found virtually tumor free after 35 d.p.i.

**Figure 4:**
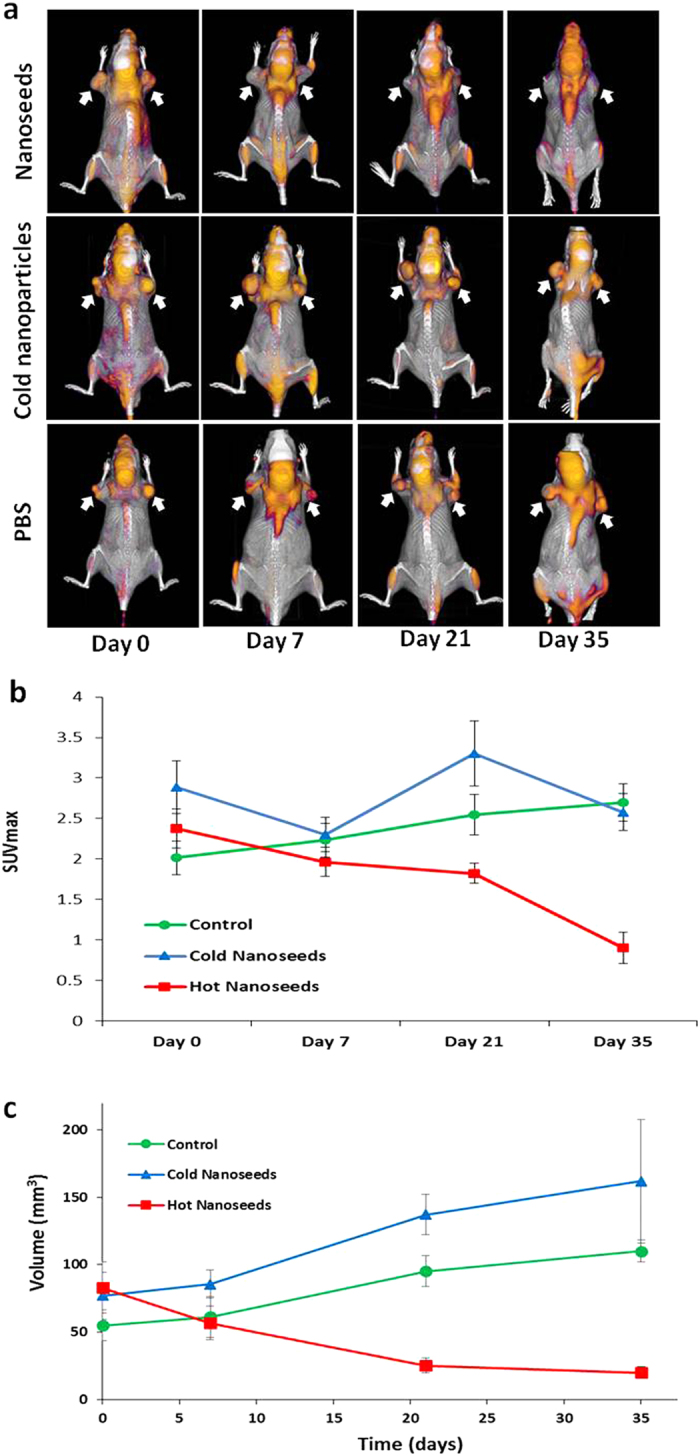
Assessment of therapeutic efficacy of ^103^Pd@Au nanoseeds in PC3 tumor bearing mice by PET/CT imaging. (**a**) Serial FDG-PET/CT images acquired at 0, 7, 21, and 35 days post intratumoral injection of PBS (upper panel), cold Pd@Au nanoparticles (middle panel), and hot ^103^Pd@Au nanoseeds (lower panel). A significant tumor FDG uptake reduction was observed in the treatment group with ^103^Pd@Au nanoseeds as compared to that in the pbs and cold Pd@Au nanoparticles treated groups. White arrows indicate tumor sites. (**b**) Quantitative PET analysis (SUV_max_ values versus time), (**c**) Comparative tumor volume changes determined by CT scan analysis (mean ± SEM).

Positron emission tomography (PET) with [^18^F]FDG (2-[^18^F]fluoro-2-deoxyglucose) was also employed for the assessment of therapeutic efficacy of the nanoseeds as it is widely used in oncology for noninvasively monitoring of patient responses to therapies^19^,^20^,^21^. Through imaging quantification of elevated glucose consumption of tumor cells, FDG-PET provides a noninvasive measure of tumor cell viability. Figure 4a shows representative FDG-PET/CT images of the three groups of tumor-bearing mice at different time points during the 5-week treatment period. Before the treatment (day 0), the three groups of mice exhibited approximately the same tumor size range and a similar level of FDG uptake in tumors. After 5 weeks of treatment, a significant tumor FDG uptake reduction was observed in the nanoseeds treated group (upper panel) as compared to that in the PBS (lower panel) and cold Pd@Au nanoparticles (middle panel) treated groups. Quantitative imaging analysis of the PET images is displayed in Fig. 4b as the maximum standardized uptake value (SUV_max_) versus time, where SUV represents the concentration of radioactivity in the tumor, normalized to the injected FDG dose and the mouse body weight. After the 5-week treatment, tumor SUV_max_ showed a 62% decrease in the mice treated with the nanoseeds from day 0 to day 35 (p = 0.00041). Impressively, it also represents 65% (p = 0.00019) and 66.5% (p = 0.00028) less uptake of FDG in tumors treated with PBS and cold Pd@Au nanoparticles, respectively. The SUV_max_ decrease in the nanoseed-treated group clearly indicates the anticipated therapeutic responses of tumors to the nanoseeds. The corresponding CT images were also utilized to determine the tumor volume. Shown in Fig. 4c, the tumor volume changes measured by CT are consistent with those obtained by caliper.

The remarkable therapeutic efficacy demonstrated here proves that our injectable nanoseeds truly have the potential to replace current millimeter-sized brachytherapy seeds. This nanoseed-based brachytherapy is much less invasive, can effectively confine the radiation inside the tumor and efficiently deliver the desired radiotherapeutic payloads to cancer cells. The key component in this approach is to use monodispersed hollow Au nanoparticles as carriers for radiation sources, which makes the size of the nanoseeds large enough so that they cannot diffuse away from the injection sites. In addition, the Au nanoparticle carrier potentially provides another advantage: acting as radiosensitizers to enhance the DNA damage by radiation. The recent theoretical modeling^22^,^23^,^24^,^25^ and *in vitro* and *in vivo* experimental^26^,^27^,^28^,^29^,^30^,^31^,^32^,^33^,^34^,^35^,^36^,^37^,^38^,^39^ results have demonstrated that Au nanoparticles can serve as novel radiosensitizing agents for external radiation therapy^26^,^27^,^28^,^29^,^30^,^31^,^32^,^33^,^34^,^35^,^36^ and brachytherapy^37^,^38^,^39^. Published work strongly suggests that Au nanoparticles can function as catalysts for the radiolysis of water under radiation exposure^40^,^41^,^42^, which generates more reactive oxygen species (ROS) inducing DNA damage or cell death. Such enhancement effect of Au nanoparticles in the nanoseeds might play a role in achieving the observed high therapeutic efficacy here, and could potentiate a combination radiotherapy methodology using nanoseed brachytherapy and external X-ray radiation therapy for an optimum therapeutic outcome.

## Conclusions

We have described a facile method to efficiently incorporate radioactive isotopes onto an inert hollow Au nanoplatform to form brachytherapy nanoseeds. Capable of being permanently retained in tumor sites after intratumoral implantation and effectively reducing tumor burden without causing adverse side effects, this type of nanoseeds is expected to find applications in anti-cancer therapies, especially for the treatment of unresectable solid tumors. Given the results presented in this work, we believe the brachytherapy approach enabled by our nanoseeds would overcome the main drawbacks of the conventional brachytherapy with significantly reduced side effects and offer earlier thus more efficacious treatment that would lead to prolonged survival and better life quality of cancer patients.

## Methods

### Animals

All animal care and experimental procedures were approved by the University of Texas Southwestern Medical Center Institutional Animal Care and Use Committee in compliance with the United States Public Health Service Standards and National Institutes of Health guidelines. All experiments were performed on SCID mice (Male, 27 ± 2 g, Age 6–8 weeks). Throughout the experiment, the animals were housed in laminar flow cages maintained at 22 ± 2 °C, 50–60% relative humidity, under a 12-hr light:12-hr dark cycle. Four rats per plastic cage were housed and allowed to acclimatize in standard conditions for one week. The rats were permitted free access to tap water and commercialized food (Jae II Chow, Korea), throughout the experiment.

### Materials

PdCl_2_ and CuSO_4_.5H_2_O were obtained from Sigma-Aldrich (St. Louis, MO) and Alfa Aesar (Ward Hill, MA), respectively. Radioactive ^103^Pd was purchased from Nordion (Ontario, Canada). PBS was purchased from Invitrogen Corporation (Carlsbad, CA). All other solvents and reagents were of analytical purity grade and were purchased from VWR (Brisbane, CA). All aqueous solutions were prepared in Millipore Milli-Q water (18 MΩ-cm) that was obtained from a Millipore Gradient Milli-Q water system (Billerica, MA).

### Synthesis of hollow gold nanoparticles (HAuNPs)

HAuNPs were synthesized using bubble template synthesis developed by us^43^. Briefly, electrochemically evolved hydrogen nanobubbles serve as templates. The high concentration of hydrogen molecules in the bubble boundary reduces the Au^+^ ion to form Au clusters. Subsequently, the metal clusters act as catalysts to trigger the autocatalytic disproportionation reaction of Na_3_Au(SO_3_)_2_, which leads to the formation of a gold shell around the hydrogen bubble. The metal Au gradually grows from clusters or particles to a porous network. Synthesized HAuNPs feature a sub-25 nm shell with a 50–70 nm hollow core. Commercial anodic aluminum oxide (AAO) membrane was used in the process, and nanobubbles and nanoparticles formed on the wall of the channels inside AAO membranes.

### Synthesis of ^103^Pd@Au nanoseeds

Each alumina membrane with a diameter of 1 cm containing trapped HAuNPs were treated with 9 ml of Cu^2+^ plating solution (containing 0.4 M CuSO_4_ in 5% *w/v* EDTA, 37% *v/v* formaldehyde and 1.0 M NaOH in 1:1:1 v/v proportion) for 20 min. After 20 minutes, the membrane containing Cu-plated HAuNPs was washed with water thrice. This membrane was first drained using a vacuum filtration setup with 0.1 M citric acid solution thrice to completely soak the channels of membrane with citric acid, and then 3 ml of 0.1 M citric acid solution containing 4.37 mCi ^103^Pd was added. Plating of ^103^Pd on HAuNPs was then continued for 24 h, followed by addition of cold Pd plating solution (containing 0.0025 M PdCl_2_ in 0.4 M citric acid solution) to replace all Cu. After 1 h, 2 M NaOH was added to dissolve the membrane and the resultant ^103^Pd@Au nanoseed suspension was washed thrice with water by centrifugation (Legend Micro 21, FL, USA) at 14000 rpm for 10 min with sonication (Branson 2510, CT, USA) after each centrifugation run. The resultant pellet of ^103^Pd@Au nanoseeds was dispersed by sonication in required quantity of PBS 7.4. The overall process yielded ^103^Pd@Au nanoseeds with >80% radiolabeling efficiency as determined by dose calibrator (Capintech Inc, PA, USA). Dynamic light scattering (DLS) assessment confirmed the synthesized ^103^Pd@Au nanoseeds to be monodisperse with mean particle size of 140.5 ± 7.6 nm.

### SPECT imaging method using the low energy emission of ^103^Pd

A SPECT imaging method with ^103^Pd was developed in a NanoSPECT/CT Plus System (Bioscan, Washington, DC, USA). ^103^Pd isotope was added to the NanoSPECT/CT Plus isotope library by setting the energy peak and width to 18 keV and 60%, respectively. Quantification calibration was performed subsequently using a 3mL syringe and 1.2 mCi of ^103^Pd.

### Intratumoral administration of nanoseeds and SPECT analysis

^103^Pd@Au nanoseed dose (≈1.5 mCi) was prepared in PBS pH 7.4 and injected intratumorally in PC3-tumor bearing SCID mice. Intratumoral injection was carefully performed at 6–9 randomly selected locations. After injection, small animal imaging was performed using NanoSPECT/CT Plus System. After the intratomor injection of each dose, SPECT and CT images were acquired at 0, 1, 2, 4, 7, 14, 21 and 35 d.p.i. The field of view (FOV) of the SPECT/CT was centered at the shoulders of the mouse. The CT imaging was performed using 360 projections per rotation with 55 kVp, 1000 ms exposure, and the binning factor of 1:1. The SPECT data were collected with 4 detector arrays collimated with multi-pinhole apertures giving a post-reconstruction resolution of 0.73 mm. The SPECT image reconstruction was carried out using HiSPECT NG (Scivis wissenschaftliche Bildverarbeitung GmbH, Germany) with 35% smoothing, 100% resolution, and 3 × 3 iterations (Standard mode). The quantification of the tumor activity was performed using the InVivoScope 2.0 software package (Bioscan, Washington, DC, USA). After co-registration of the CT and SPECT images, a cylindrical region of interest (ROI) was drawn, encompassing the tumor and liver in all planes containing the organs.

### FDG-PET/CT imaging to assess the efficacy of ^103^Pd@Au nanoseeds

Mouse PET/CT imaging was performed using Siemens Inveon PET/CT multimodality system (Siemens Medical Solutions, Knoxville, TN) with effective spatial resolution of 1.4 mm at the center of field of view (FOV). All animals were fasted for 12 hours prior to PET imaging. Each mouse received 150 uCi of FDG in 150 uL in saline intravenously via tail vein injection. The mice were placed on a heat pad before and during image acquisition. PET images were acquired one hour post-injection (P.I.), for 15 minutes, with animals under 2.5% Isoflurane. PET images were reconstructed into a single frame using the 3D Ordered Subsets Expectation Maximization (OSEM3D/MAP) algorithm. CT images were acquired immediately after PET with the FOV centered at the shoulder of the mouse. CT projections (360 steps/rotation) were acquired with a power of 80 kVp, current of 500 μA, exposure time of 145 ms, binning of 4, and effective pixel size of 102 μm. The CT reconstruction protocol used a downsample factor of 2, was set to interpolate bilinearly, and used a Shepp-Logan filter. PET and CT images were co-registered in Inveon Acquisition Workplace (Siemens Medical Solutions, Knoxville, TN) for analysis. Regions of interest (ROI) were drawn manually, encompassing the tumor in all planes containing the tissue. The target activity was calculated as percentage injected dose per gram.

### *Ex vivo* measurements of radioactivities and Au and Pd contents among various organs

At 1 day, 1 week, 2 weeks, 3 weeks, and 5 weeks after the injection of ^103^Pd@Au nanoseeds, three mice were sacrificed and the desired organs including blood, heart, lung, muscle, bone, fat, liver, spleen, kidney, stomach, small intestine, large intestine, brain, tail, and tumor were collected, weighed and transferred to 20 ml vials. To measure the radioactivity associated with each organ, the activity of each vial was measured in a γ-counter (Perkin Elmer 2480 Wizard) and recorded as counts per minute. Then, aqua regia was added to the vials and left overnight to digest the organs. After 24 h, the aqua regia is boiled off at 150 °C. After boiling, 10 ml of 1% HCL solution was added to vials, in which they were then sonicated for 30 minutes. The Au and Pd concentration were then measured in an inductively coupled plasma mass spectrometer (ICP-MS, Agilent 7700×). The measurement was repeated at least three times for each sample.

### Statistical analysis

Quantitative data were expressed as mean ± standard errors of mean (SEM). Comparison among the means and the significance evaluation were performed by one-way ANOVA, where *P* values of <0.05 were considered statistically significant. The data from different groups and within each individual group at different time points were compared to determine whether they are statistically distinguishable. All data analysis was carried out using SPSS Ver. 16.0 software (IBM SPSS Statistics).

## Additional Information

**How to cite this article**: Moeendarbari, S. *et al.* Theranostic Nanoseeds for Efficacious Internal Radiation Therapy of Unresectable Solid Tumors. *Sci. Rep.* **6**, 20614; doi: 10.1038/srep20614 (2016).

## Supplementary Material

Supplementary Information
