# Supplementary material for: Theranostic Nanoseeds for Efficacious Internal Radiation Therapy of Unresectable Solid Tumors
Source: Sci Rep. 2016 Feb 8;6:20614. doi: 10.1038/srep20614 (PMC4745015; doi:10.1038/srep20614)
Supplement: Supplementary Information [file srep20614-s1.pdf]

Theranostic Nanoseeds for Efficacious Internal Radiation Therapy of Unresectable Solid Tumors

Sina Moeendarbari[1], Rakesh Tekade[2], Aditi Mulgaonkar[2], Preston Christensen[2], Saleh Ramezani[2], Gedaa Hassan[2], Ruiqian Jiang[1], Orhan K. Öz[2], Yaowu Hao[1*], Xiankai Sun[2,3*]

[1] Department of Materials Science and Engineering, University of Texas at Arlington, TX 76019, USA

[2] Department of Radiology and [3] Advanced Imaging Research Center, University of Texas Southwestern Medical Center, Dallas, TX 75390, USA

** Corresponding authors:* Xiankai.Sun@UTSouthwestern.edu, yhao@uta.edu

Supplementary Information

Ex vivo studies on the biodistribution of the nanoseeds

The biodistribution of the nanoseeds were investigated by an *ex vivo* assay At different time points during our study (1 day, 1 week, 2 weeks, 3 weeks, and 5 weeks after injection), three mice treated with nanoseeds were sacrificed and the organs of interest (blood, heart, lung, muscle, bone, fat, liver, spleen, kidney, stomach, small intestine, large intestine, brain, tail, and tumor) were excised, weighed, and then transferred to 20 ml vials. To measure the radioactivity associated with each organ, the activity

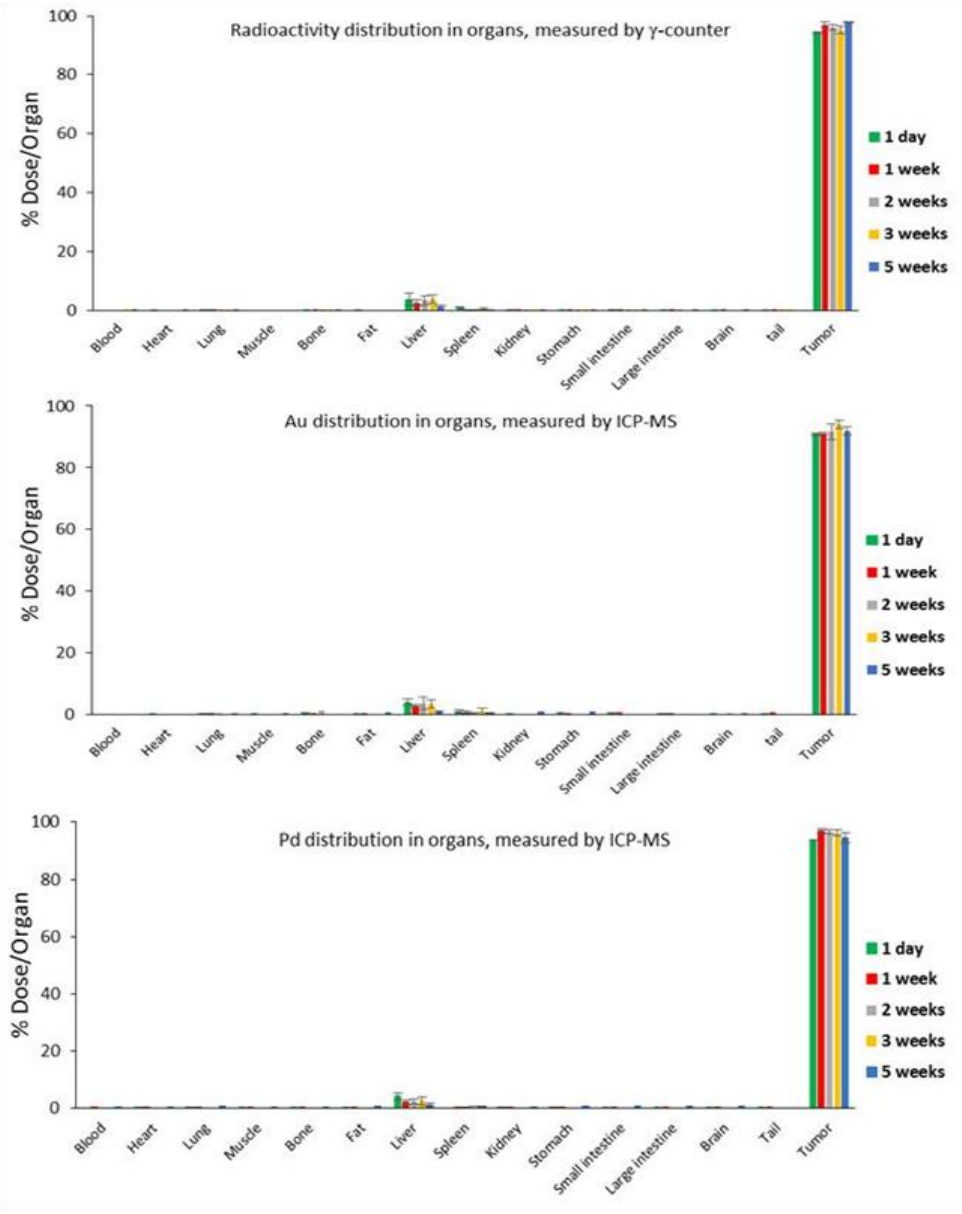

Figure S1. Retention of (a) radioactivity, (b) Au, and (c) Pd content in mice from 1 day to 5 weeks after the injection of the nanoseeds (means ± SEM).

of each vial was measured in a γ-counter (Perkin Elmer 2480 Wizard) and recorded as counts per minute. Then, aqua regia was added to the vials and left overnight to digest the organs. After 24 h, the aqua regia

is boiled off at 150°C. After boiling, 10 ml of 1% HCL solution was added to vials, in which they were then sonicated for 30 minutes. The Au and Pd concentration were then measured in an inductively coupled plasma mass spectrometer (ICP-MS, Agilent 7700x). The measurement was repeated at least three times for each sample. The obtained biodistribution data are shown in Figure S1. As can be observed, there is a good consistency between the γ-counter and ICP-MS results (no statistically significant difference, $p = 0.88$), indicating that the radioactive isotopes of ^{103}Pd stayed with the nanoseeds during the five weeks of our therapeutic study. The *ex vivo* biodistribution study demonstrated that 95.19 ± 0.94 % of the nanoseeds remained inside the tumor, while 3.31 ± 1.11 % and 0.39 ± 0.24 % went to the liver and spleen, respectively. . No meaningful uptake was observed in other tissues. It is clear that the tumor uptake was maintained essentially the same ($p = 0.35$) over the five weeks of the study.

Toxicity Studies

Liver and kidney are two key organs that are involved in uptake and localization of gold nanoparticles based delivery systems, and hence are first line of vital organs that may be affected by the ^{103}Pd@Au nanoseeds radiotherapy. Biocompatibility is a vital function that must be completely established to tag Generally Regarded as Safe (GRAS) tag for the successful transformation of any developed therapeutics to clinic.

Toxicity of ^{103}Pd@Au nanoseeds based radiotherapy was assessed and compared with control as well as cold HAuNS treated groups. Briefly, PC3 tumor bearing mice were divided in three groups (n=5). Group-I was treated with PBS pH 7.4, while group-II and II comprised of animal subjects treated with cold Pd@Au nanoparticles and nanoseeds treated animals. Following treatments, complete blood analysis, Alanine transaminase (ALT), Aspartate transaminase (AST), Blood urea nitrogen (BUN) and creatinine levels were monitored at 10 and 30 d.p.i.

BUN is a common blood test to reveal how well kidneys and liver are working. A BUN test measures the amount of urea nitrogen in the blood. A higher BUN level indicated abnormality in kidneys or liver. BUN test performed on control, cold nanoparticles treated group as well as nanoseeds treated groups showed an insignificant change in blood associated nitrogen suggesting the therapy to be eliciting no side effects on liver or kidney.

Creatinine is removed from the body entirely by the kidneys and determination of creatinine levels in blood can clearly reveal how well kidneys are working. If kidney functions abnormally then creatinine level increases in the blood due to less release of creatinine through urine. Hence, kidney function as additionally assessed by determining the creatinine levels in the blood., creatinine levels in nanoseeds treated animal subjects was found to be of similar to that of control subjects. This outcome is in agreement with BUN assay that confirms that nanoseeds based radiotherapy elicits no kidney related toxicity.

AST is an enzyme that helps metabolize alanine, an amino acid. An increase in AST levels may indicate liver damage or disease. AST levels in nanoseeds treated groups increased 10 d.p.i and 30 d.p.i.; however, this increase was found to be age related as similar trend of AST levels was found in both control, cold nanoparticles treated groups. To further confirm liver functioning ALT assay was also performed.

ALT is an enzyme found in the liver that helps your body metabolizes protein. When the liver is damaged, ALT is released into the bloodstream and levels increase. No notable change in ALT level was found in nanoseeds treated groups after 10 d.p.i and 30 d.p.i., and the levels were found to be similar to that of untreated control groups at all assessed time points. After 10 d.p.i, no significant alternation in ALT level was found in nanoseeds treated group (25.17 ± 3.90 U/L) compared to control (26.0 ± 6.71 U/L) and cold nanoparticles treated group (28.27 ± 9.6 U/L) treated subjects. Further, similar ALT levels were observed in nanoseeds treated groups after 30 d.p.i that confirmed that no immediate or delayed liver toxicity is associated with nanoseeds based radiotherapy.

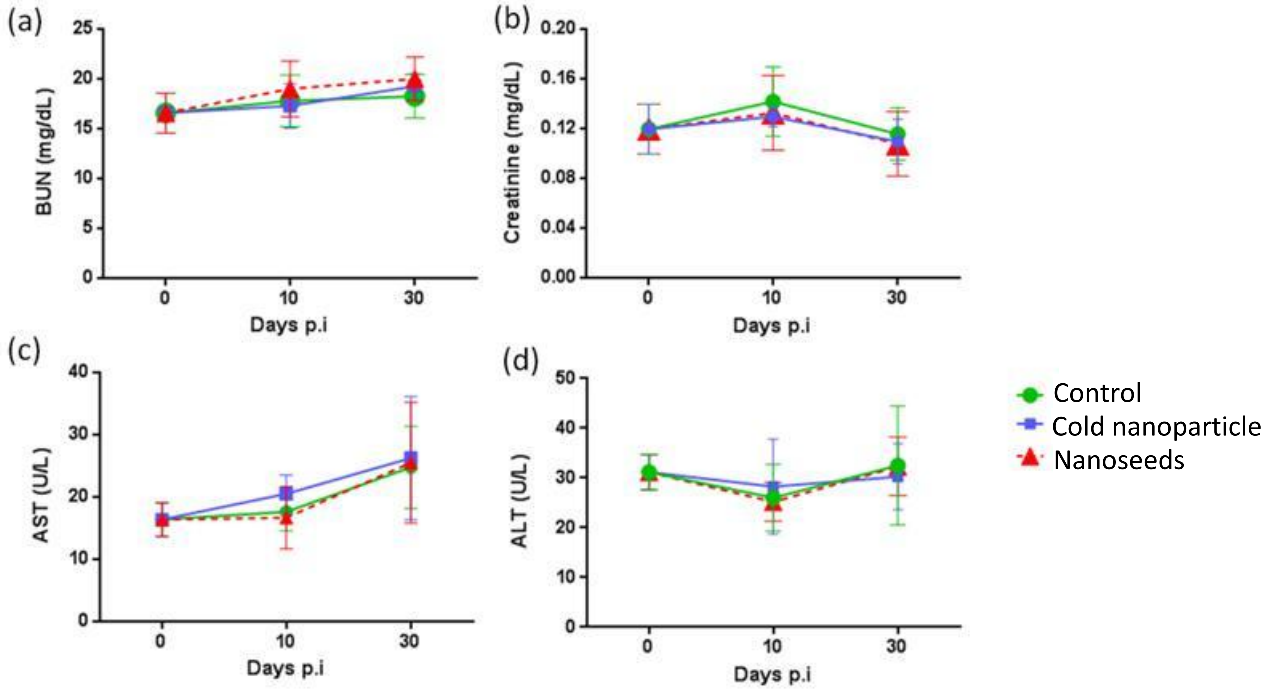

Figure S.2. (A) BUN, mg/dl, (b) creatining mg/dL, (C) AST, U/L, and (d) ALT, U/L levels in control, cold nanoparticle treated and nanoseeds treated mice.

The utility of the complete blood count (CBC) is serving as a major tool to aid diagnosis and monitor disease progression as well as therapy related toxicity. CBC analysis was done on control, cold nanoparticles and nanoseeds treated groups. Red blood cell (RBC) count as well as mean hemoglobin volume per RBC (MCH) remains unaffected throughout the study suggesting that the therapy is not resulting into any hemolytic effect.

Notably, ^{103}Pd-HAuNS radiotherapy initially reduced the white blood cell (WBC) count 10 d.p.i., which is common to all radiotherapy. However, the effect was found to be reversible, while recovering the normal WBC count to normal after 30 d.p.i. Similar effect was observed in case of platelet counts, which also decreased following the nanoseeds implantation and recovered to same level that of control 30 d.p.i. Basophil count in control and nanoseeds treated group was found to be insignificantly differing from each other at 0, 10 and 30 d.p.i., indicating that the fluctuation is cancer and not the therapy related. After nanoseeds implantation, neutrophil count increased, while eosinophil count decreased initially after 10 d.p.i compared to control, which is an inherent side effect of radiotherapy. However, this was found to be temporary and started to regain to original value when measure after 30 d.p.i.

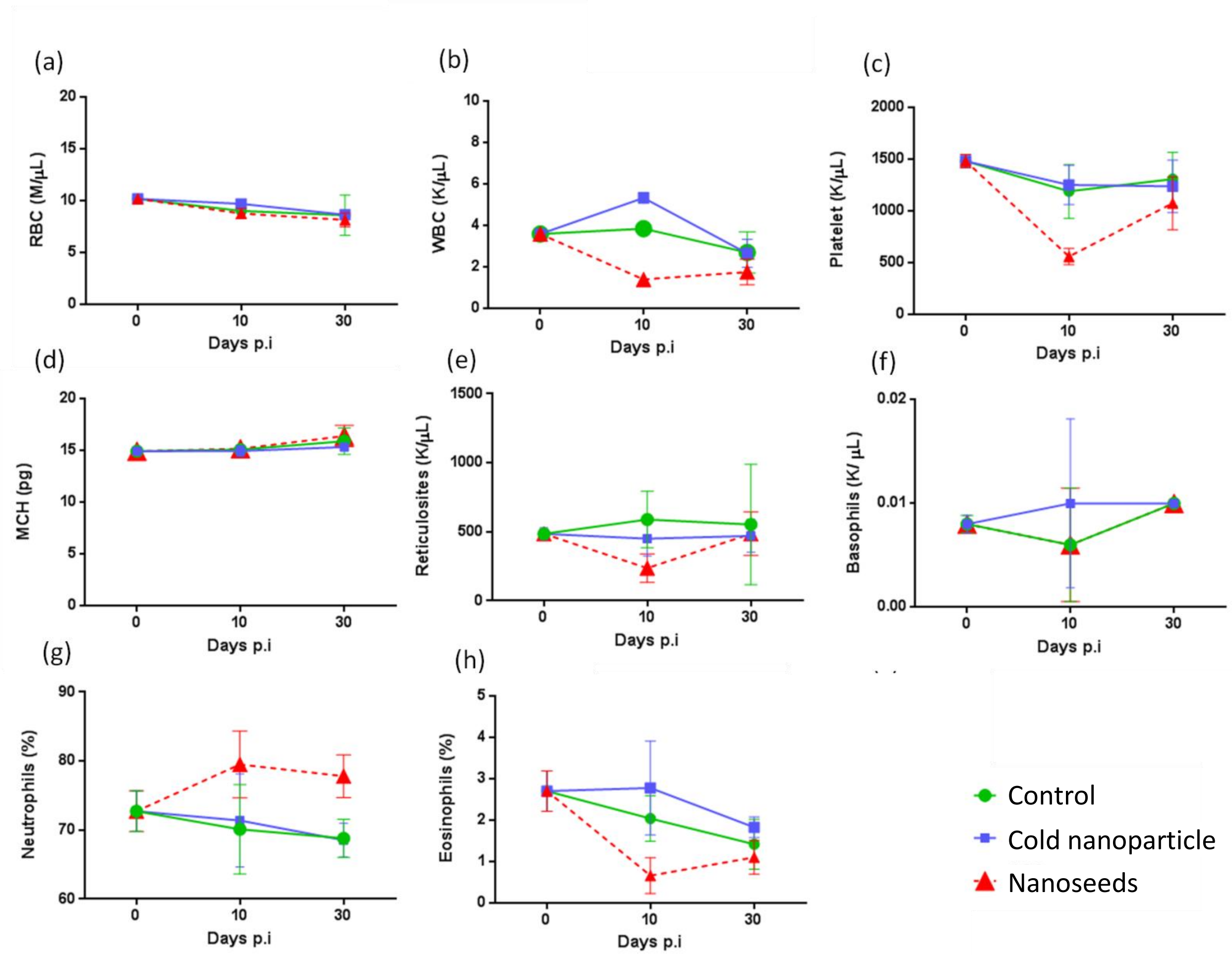

Figure S3. (a) RBC m/μL, (b) WBC, K/ μL, (c) platelet count, K/ μL, (d) MCH. Pg, (e) reticulosites, K/ μL, (f) basophils, K/ μL, (g) Neutrophils, %, (h) Eosinophils, % in control, cold nanoparticles treated and nanoseeds treated mice.
